# Pharmacogenetic drug use in young danish individuals with severe mental disorders

**DOI:** 10.1192/j.eurpsy.2021.394

**Published:** 2021-08-13

**Authors:** C. Lunenburg, K. Ishtiak-Ahmed, T. Werge, C. Gasse

**Affiliations:** 1 Dep. Affective Disorders, Aarhus University Hospital Psychiatry, Aarhus N, Denmark; 2 Department Of Clinical Medicine, Aarhus University, Aarhus, Denmark; 3 Institute Of Biological Psychiatry, Mental Health Center Sct. Hans, Copenhagen, Denmark; 4 Psychosis Research Unit, Aarhus University Hospital Psychiatry, Aarhus N, Denmark; 5 Centre For Integrated Register-based Research, Aarhus University, Aarhus, Denmark

**Keywords:** Pharmacogenetics, Drug Utilization, severe mental disorders

## Abstract

**Introduction:**

Pharmacogenetics (PGx) studies genetic variance and related differences in drug outcomes. PGx guidelines for psychotropic drugs are available (PGx drugs), but PGx testing is used only limitedly in psychiatric clinical practice.

**Objectives:**

The aim of this study is to pinpoint different aspects of PGx drug use in the population, to support clinical uptake of PGx.

**Methods:**

This drug utilization study investigated prescription PGx drug use in 56,065 young individuals with different severe mental disorders (SMD) in the Danish iPSYCH sample (born 1981-2005). We investigated the number of PGx drug users (incidence, prevalence), age (at first PGx drug use), sex, multiple PGx drugs per user (in light of panel-based PGx testing) and concomitant use of PGx drugs (in light of combinatorial PGx testing).

**Results:**

We identified substantial PGx drug use in terms of incidence rates (e.g. 333 per 10,000 person years for the anticonvulsant lamotrigine) and prevalence (e.g. 15,260 users for the antidepressant citalopram) in patients with SMD. The age of first time PGx drug use ranged from 11.6-20 years, depending on SMD and sex. On average, more than one PGx drug was used by a single person (range 1.6-5.6 drugs, depending on SMD) or even used concomitantly (41-69%) affecting mostly two different PGx genes (84-92% of concomitant PGx drug users).

**Conclusions:**

PGx drugs were frequently used in young individuals with SMD, often subsequently and concomitantly, arguing for panel-based/combinatorial PGx testing over single-gene testing. PGx testing could be applied already at a very young age.

**Disclosure:**

We thank the iPSYCH consortium, in specific the iPSYCH PI’s (Merete Nordentoft, Anders Børglum, Preben B. Mortensen, Ole Mors, Thomas Werge and David M. Hougaard). The iPSYCH project is funded by the Lundbeck Foundation Denmark and the universities and un

